# In the Mind of the Market: Theory of Mind Biases Value Computation during Financial Bubbles

**DOI:** 10.1016/j.neuron.2013.07.003

**Published:** 2013-09-18

**Authors:** Benedetto De Martino, John P. O’Doherty, Debajyoti Ray, Peter Bossaerts, Colin Camerer

**Affiliations:** 1 Division of the Humanities and Social Sciences, California Institute of Technology, Pasadena, CA 91125, USA; 2 Department of Psychology, Royal Holloway University, London TW20 0EX, UK; 3 Computation and Neural Systems, California Institute of Technology, Pasadena, CA 91125, USA; 4 Department of Finance, David Eccles School of Business, University of Utah, Salt Lake City, UT 84112, USA

## Abstract

The ability to infer intentions of other agents, called theory of mind (ToM), confers strong advantages for individuals in social situations. Here, we show that ToM can also be maladaptive when people interact with complex modern institutions like financial markets. We tested participants who were investing in an experimental bubble market, a situation in which the price of an asset is much higher than its underlying fundamental value. We describe a mechanism by which social signals computed in the dorsomedial prefrontal cortex affect value computations in ventromedial prefrontal cortex, thereby increasing an individual’s propensity to ‘ride’ financial bubbles and lose money. These regions compute a financial metric that signals variations in order flow intensity, prompting inference about other traders’ intentions. Our results suggest that incorporating inferences about the intentions of others when making value judgments in a complex financial market could lead to the formation of market bubbles.

## Highlights

• Theory of mind can be maladaptive in complex institutions like financial markets
• Dorsomedial prefrontal cortex biases signals in ventromedial prefrontal cortex
• Both regions compute financial metric signals
• Incorporating inferences about the intentions of others could lead to market bubbles

## Teaser

De Martino demonstrates that the ability to infer the intentions and mental states of other individuals (“theory of mind”) biases evaluation when people interact with complex modern institutions like financial markets, contributing to the formation of economics bubbles.

## Introduction

In February 1637 in Amsterdam, the cost of a single exotic tulip bulb reached a price equal to ten times what a skilled craftsman earned in a year. The price of the same bulb collapsed a few days later. The dramatic rise and fall of tulip bulb prices is a famous historical example of a financial bubble (Kindleberger and Aliber, 2005). A bubble is conventionally defined by active trading of an asset at prices that are considerably higher than its intrinsic fundamental value. Examples of modern bubbles include Japanese stocks in the 1990s, the US high-tech sector in the late 1990s, and housing prices, which rose and crashed in many countries from 2000–2008. All of these bubbles (especially the housing crash) caused long-lasting macroeconomic disruptions (Shiller, 2005).

Modern bubble episodes have also led to a substantial shift in thinking about the capacity of prices to act as sober information aggregation mechanisms that guide efficient allocation of capital. Policy makers, academics, and market participants alike are now more familiar with, and groping to understand, the ways that prices can reflect pathological valuation and are actively debating whether policy interventions can help (Akerlof and Shiller, 2009).

Despite these dramatic historical and modern examples, there is no well-accepted theory of how bubbles start and end. One common definition of bubbles is rapid price appreciation followed by a crash (Brunnermeier, 2008). However, this definition has no predictive power for identifying an ongoing bubble, since it does not identify a bubble before it crashes. Furthermore, fundamental asset values are rarely known with precision, so it is difficult to identify a bubble if bubbles are defined as prices above an elusive fundamental value.

One way to learn about bubbles is to observe trading in an experimental market for artificial assets that have a known fundamental value. In these markets, price variation cannot be explained by changes in fundamentals. In fact, several carefully controlled economics experiments have shown that certain classes of asset markets do generate price bubbles quite regularly, even when intrinsic values are easy to compute and are known to traders (Smith et al., 1988; Camerer and Weigelt, 1993; Porter and Smith, 2003; Lei et al., 2004). The nature of bubbles has also been intensely investigated in theory (Abreu and Brunnermeier, 2003; Yu and Xiong, 2011), but empirical reasons why bubbles arise and then crash are still not well understood in economics (Xiong, 2013).

Recent work in neuroeconomics has shown how financial decision theory can be informed by neuroscientific data (Bossaerts, 2009). In particular, studies have started to dissect the neural mechanisms by which risk processing (Preuschoff et al., 2008), anticipatory affect (Knutson and Bossaerts, 2007; Kuhnen and Knutson, 2005), fictive learning signals (Lohrenz et al., 2007), inference about information possessed by other traders (Bruguier et al., 2010), and mental accounting of trading outcomes (C. Frydman, personal communication) shape financial decisions. However, the neural mechanisms underpinning the formation of a financial bubble are still unknown. Understanding of these mechanisms could prove critical in distinguishing between alternative hypotheses, each requiring different macroeconomic interventions.

This study, which combines experimental finance settings together with behavioral modeling and neuroimaging methods, aims to identify the neural coding scheme at the core of bubble formation. We focus here on how the representation of assets trading values in ventromedial prefrontal cortex (vmPFC), a brain region heavily involved in representing goal value (Rangel et al., 2008; Boorman et al., 2009; Chib et al., 2009; Hare et al., 2009; Levy and Glimcher, 2012), are modulated by formation of a bubble. Our hypothesis is that the increase in prices observed in bubble markets is associated with the neural representation of inflated trading values in vmPFC, which produces an enhanced susceptibility to buying assets at prices exceeding their fundamental value. We test the hypothesis that the inflated values are caused by participants’ maladaptive attempts to forecast the intentions of other players in a fast-growing market. In particular, we propose that the more dorsal portion of the prefrontal cortex (dmPFC), a region well known to represent the mental state of other individuals (also known as theory of mind; ToM) (Frith and Frith, 2003; Amodio and Frith, 2006; Hampton et al., 2008), is involved in updating the value computation in vmPFC, stimulating the formation of a financial bubble. In order to clarify the role played by intentions in modulating activity in these brain regions during financial bubbles, we introduce a computational concept from financial theory. This metric captures the dynamic changes from a steady, regular arrival of buying and selling orders to a more variable arrival process (perhaps signaling the start of a bubble, as orders arrive rapidly due to excitement, or an impending crash, when orders arrive slowly as traders hold their breath) that can signal the presence of strategic agents in a market. Activity in medial prefrontal regions is correlated with this index more strongly in bubble markets than in nonbubble markets and is associated with the individual’s propensity to ride the financial bubble.

## Results

### Experimental Markets

Twenty-one participants were scanned while trading in experimental markets. Trading activity in six actual experimental markets (collected in previous behavioral studies; Porter and Smith, 2003) was replayed over a 2-day scanning schedule. On each day, the participants traded in three experimental markets. Each market was divided into fifteen trading periods. During each trading period, the scanned participants observed a fast-motion visual representation of the prices of offers to sell (asks) and offers to buy (bids), which were actually inputted by the participants who had taken part in the original behavioral experiments.

Subjects started with a cash endowment of $60. The screen was frozen at random intervals (2–3 times each period). At these freeze points, participants were allowed to stay (do nothing) or buy or sell one, two, or three shares at the current market price by pressing a keypad. After the choice was inputted, an update of the participants’ portfolio (number of the shares held and cash) was presented on the screen. This was followed by a variable resting phase. At the end of each of the fifteen periods, the trading activity was interrupted, and participants were shown the dividend paid to the shareholder for that period.

The traded assets paid a dividend worth an expected value of $0.24 in each period to subjects who held those assets. Therefore, the intrinsic expected value of buying and holding assets was initially $3.60. The assets’ intrinsic value (fundamental value) declined by $0.24 after each period (since there were fewer future dividends lying ahead). The asset value in period t was therefore $0.24 × (15 − t + 1) (see Experimental Procedures for more details).

**Figure 1:**
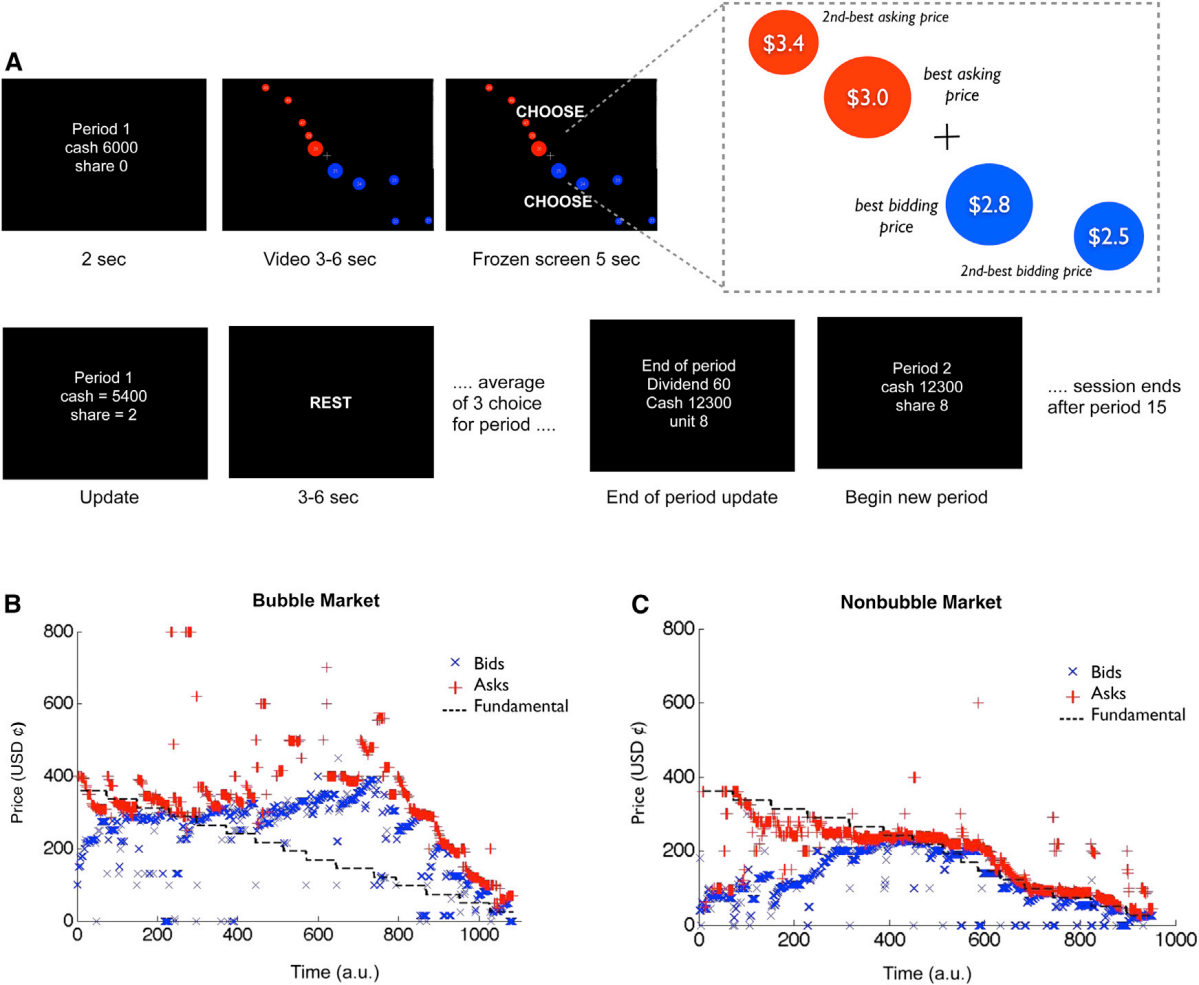
Task (A) Structure of the task: at the beginning of each period, participants were shown a message stating the period number and the value of their portfolio (shares and cash). This was followed by a video showing an intuitive graphical replay of the order (asks and bids) and trade flow. The orders were arranged by price level (see illustrative diagram on the right corner). Whenever a trade occurred, the best bid (if a sale) or best ask (if a purchase) briefly (0.5 s) changed color to green, after which the circle disappeared. The circles constantly rearranged to ensure that the best bid and ask circles were closest to the midpoint of the screen (for more details see Bruguier et al., 2010). After a variable time interval (3–6 s), the screen was frozen for 5 s, and subjects used their initial endowment of $60 to either buy or sell (1, 2, or 3 shares) or stay by pressing a keypad. At the end of the choice period, an update screen summarized their current portfolio (i.e., cash and shares). This was followed by a resting period (3–6 s). At the end of each period (15 periods in total), a dividend was randomly extracted from (0*¢* 8*¢* 28*¢* 60*¢*), and subjects paid for the number of shares held (in the case of short selling, subjects had to pay the cost of the dividend for the number of negative shares held). The dividend for that period was displayed to the subjects with an update of their portfolio. (B and C) Asks (red) and bid (blue) plotted against the fundamental prices (dotted line) for one of the three nonbubble markets (B) and one of the three bubble markets (C) replayed during the experiment. In the nonbubble markets condition (B), asks and bids track the fundamental price over time, while in the bubble markets condition (C), asks and bids deviate from the fundamental prices. All six of the markets used in the study are plotted in the Supplemental Information.

Three of the six sessions used in the study were nonbubble markets; in those sessions, the market prices were tracking the fundamental value of the asset closely (Figure 1A). The other three sessions were bubble markets, in which market prices rose well above the intrinsic value in later periods (Figure 1B; Figure S1 available online).

### Behavioral Results

Our initial approach was to quantify how participants’ choices (i.e., buy, sell, or stay) were influenced by market parameters such as bid and ask prices and fundamental values. We performed an ordered logistic regression using participants’ choices (i.e., buy, sell, or stay) as dependent variables and market prices and fundamental values as independent variables. The parameter estimates showed that in both the bubble and nonbubble markets, the participants’ behavior was significantly modulated by prices and fundamental values, but that those two factors explained less variance in the bubble markets data (pseudo R^2^ = 0.27; Bayesian information criterion [BIC] = 2,089) than in nonbubble (pseudo R^2^ = 0.33; BIC = 1,840). Notably, there was a significant difference between bubble and nonbubble market coefficients computed for prices (t test: t = 3.48; p < 0.05) and for fundamental value (t test: t = 4.24; p < 0.001). Coefficients for prices and fundamentals together with a summary statistics are presented in Table 1. These results suggest that during financial bubbles, participants’ choices are less driven by explicit information available in the market (i.e., prices and fundamentals) and are more driven by other computational processes, perhaps imagining the path of future prices and likely behavior of other traders.

To further investigate this issue, we measured how the neural representation of value changes when participants trade assets in bubble markets compared with nonbubble markets (using fMRI). Our hypothesis was that the increased trade volume in bubble markets should be associated with an inflated representation of portfolio profits. We reasoned that if the formation of bubbles is a consequence of inflated value representation, a brain region that codes for parametric changes in trading values should have increased activity when participants trade in bubble markets.

### Value Computation

**Figure 2:**
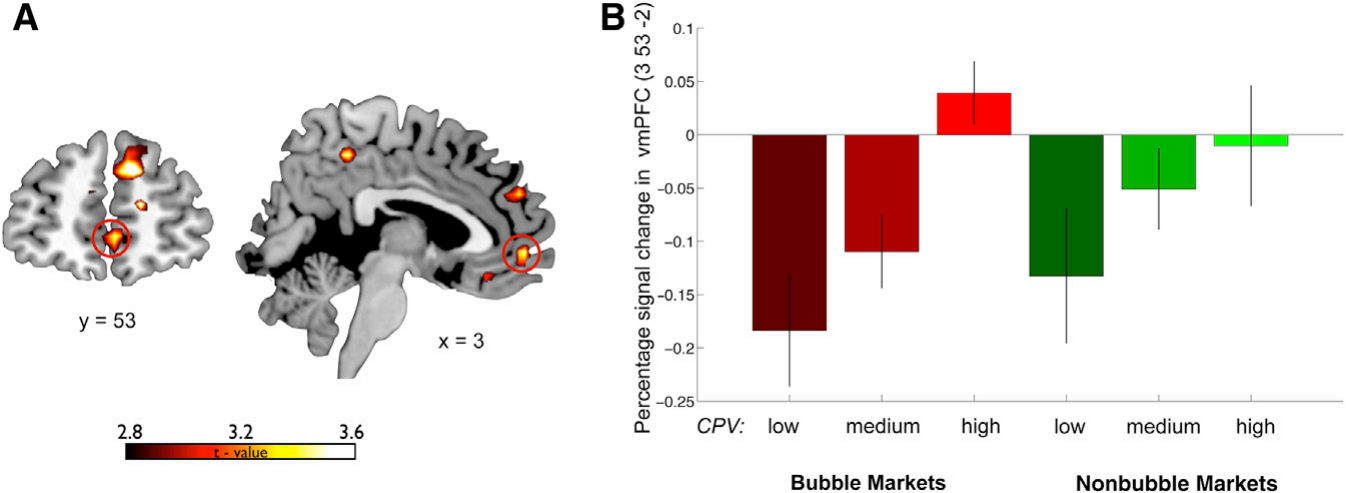
Value Signals in vmPFC (A) Increased response to parametric changes in CPV in bubble markets versus nonbubble markets. vmPFC (peak [x, y, z] = [3, 53, 2]; Z = 3.02; p < 0.05 small volume FWE corrected) representation of trading value is positively modulated in bubble markets. (B) Bar plot for the vmPFC response for three levels of CPV (low, medium, high) for bubble markets (red) and nonbubble markets (green). Note that the bar plot is shown solely for illustrative purposes (to clarify the signal pattern in vmPFC) and is not used for statistical inference (which was carried out in the SPM framework).

To test this hypothesis, we constructed a parametric variable that captured the trial-by-trial variance in the value of each participant’s trading position. We called this variable current portfolio value (CPV), a combination of the value in cash and in shares held by a participant (or trader) at each point in time (CPV[t] = cash + [shares × fundamental value at time t]). CPV was used as a parametric regressor in a general linear model to isolate changes in blood-oxygen-level-dependent (BOLD) signal underpinning the increased representation of trading values during bubble markets compared to nonbubble markets. This analysis yielded a significant interaction in ventromedial prefrontal cortex (vmPFC peak [3, 53, −2], t = 3.48; p < 0.05 small volume correction [SVC] for multiple comparison), a brain region that plays a key role in encoding the goal values that are used to guide choice (Figure 2A; for a complete list of activations see also Figure S1). We therefore confirmed, consistent with our initial hypothesis, that the parametric representation of the portfolio value (CPV) was increased during bubble markets. This is illustrated by the pattern of activity in vmPFC (percent BOLD signal changes) in response to increasing levels of CPV in both bubble and nonbubble markets (Figure 2B).

We next reasoned that if inflated trading values represented in vmPFC play a role in the formation of a financial bubble, activity in this region should predict the behavioral tendency to buy shares when their prices are above the fundamental values (a behavior that stimulates and sustains the formation of a financial bubble). To test this, we constructed an independent parameter that quantified the participants’ tendency to ride the bubble. We called this between-subject index “bubble susceptibility,” which is the extra price paid by participants to purchase shares at prices above the fundamental value (see Experimental Procedures for more details).

**Figure 3:**
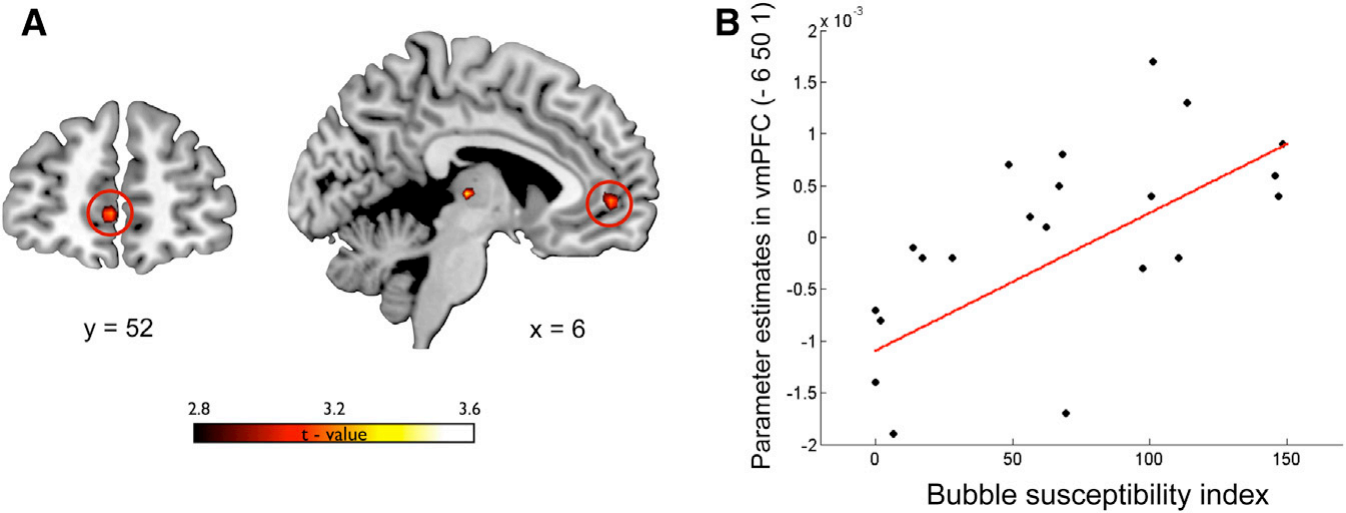
Bubble Susceptibility Index (A) Activity in vmPFC is positively modulated by the individual propensity to ride a financial bubble. Between-subject regression analysis entering the bubble susceptibility index (i.e., the extra price paid by participants to purchase shares at prices above the fundamental value during the whole experiment) as a covariate for the increase in CPV response during bubble markets in vmPFC (peak [x, y, z] = −6, 50, 1; Z = 3; p < 0.05 small volume FWE corrected). (B) Scatter plot showing the parameter estimates for each participant. Note that the scatter plot is shown here solely for illustrative purposes (e.g., absence of outliers), and it is not used for statistical inference (which was carried out in the SPM framework).

We then entered this bubble susceptibility index as a between-subjects covariate in the parametric general linear model (GLM) model described above. This analysis yielded a significant correlation in vmPFC (peak [−6, 50, 1]; t = 3.44; p < 0.05 SVC for multiple comparisons). More precisely, activity in vmPFC was a significant predictor of the behavioral tendency to ride bubbles (Figure 3). Note that while the overall buying at prices above the fundamental value was a relatively rare phenomenon (see Figures S2 and S3), riding the bubble (in the context of our experimental setup) was clearly a suboptimal behavior, as demonstrated by the fact that those participants with high susceptibly to ride the bubble had significantly lower monetary earnings (p = 0.02), an effect due to only trading in bubble markets (nonbubble markets: p > 0.1; bubble markets: p = 0.005). Critically, low monetary earnings did not directly correlate with activity in vmPFC (p = 0.19), excluding the possibility that the correlation we identified in this region reflected increasing susceptibility to reduced earnings (independent of bubble susceptibility).

### Theory of Mind

Our next step was to investigate the mechanism causing the inflation in value representation observed in vmPFC during financial bubbles. The key difference between nonbubble markets and bubble markets is that in nonbubble markets, the value of a share is only determined by the fundamental value of the asset, while in bubble markets, profitable trading depends on accurately judging the intentions of other players in the market. Therefore, we hypothesized that the increase in value representation during a bubble market was a consequence of the fact that traders use inferences about the intentions and mental states of other agents to update their value representation. This hypothesis was supported by the fact that in our whole-brain analysis, together with increased activity in vmPFC, we isolated a network of brain regions that have previously been associated with theory of mind (Siegal and Varley, 2002; Frith and Frith, 2006; Saxe, 2006), such as temporoparietal junction (L-TPJ; [−48, −52, 25], t = 3.68), precuneus ([6, −43, 49], t = 4.9), and dorsomedial PFC (dmPFC; [9, 50, 28], t = 3.47) (Figure 3A; for a complete list of activations see also Table S1).

In particular, we focused on dmPFC because convergent evidence suggests that this region of the prefrontal cortex plays a primary role in human ability to make inferences about the mental states (including intentions) of other agents (Siegal and Varley, 2002; Amodio and Frith, 2006), enabling strategic thinking (Hampton et al., 2008). Furthermore, a previous study has shown that in experimental financial markets, activity in this area correlates with participants’ ability to predict price changes in markets due the presence of informed insider traders in the market (Bruguier et al., 2010).

**Figure 4:**
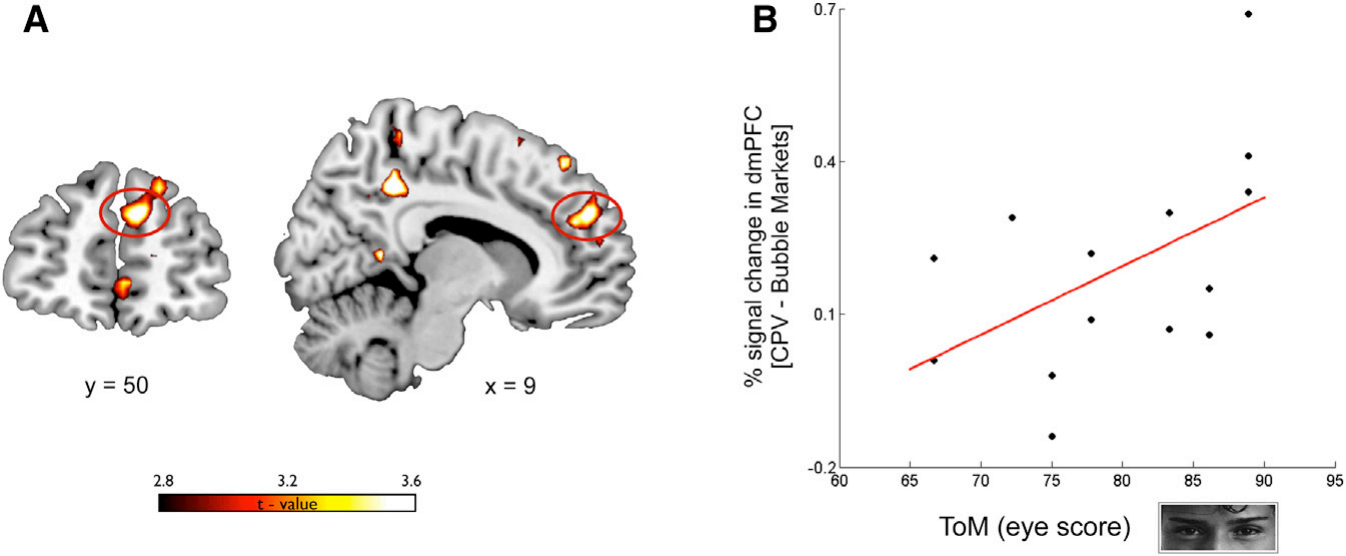
ToM Signals in dmPFC (A) Increased response to parametric changes in CPV in bubble markets versus nonbubble markets. dmPFC (peak [x, y, z] = 9, 50, 28; Z = 3.44; p < 0.05 small volume FWE corrected) is positively modulated in bubble markets. (B) Percentage of signal change extracted in this region (8 mm sphere) during bubble markets positively correlates (Spearman rank correlation coefficient ρ = 0.57; p < 0.05) with the ToM eye score collected for a subset of participants (n = 14) in a subsequent behavioral study. Notably, no significant correlation between ToM score and activity in dmPFC was isolated in nonbubble market conditions (ρ = 0.32; p > 0.1).

If activity isolated in dmPFC during bubble markets reflected mentalizing ToM activity, then we would expect a measure of neural signal change in that region during bubble markets to be associated with individual-specific measures of ToM. To test this hypothesis further, we retested a subset of participants (n = 14) who had originally participated in the bubble experiment using an online version of the eye gaze test to assess their ToM skills (Baron Cohen et al., 2001). In this task, participants looked at eye gazes and picked one of four terms that best described the mental state of the person whose eyes were shown (see Experimental Procedures). The task has correct answers, from which we constructed an index of the ToM ability of each participant. We then extracted the percentage of signal change in dmPFC in response to CPV during bubble markets (in the 8 mm sphere centered at [9, 50, 28]) for each subject and found a substantial correlation between that signal change and each subject’s ToM ability index (Spearman rank correlation coefficient ρ = 0.57; p < 0.05) (Figure 4). Critically, no significant correlation between dmPFC signal and the ToM index was found during nonbubble markets (ρ = 0.32; p > 0.1). Furthermore, we repeated the same analysis in vmPFC (in the 8 mm sphere centered at [3, 53, −2]), which showed that activity in vmPFC did not correlate with performance in the ToM task in either the bubble (ρ = 0.06; p > 0.5) or the nonbubble markets (ρ = 0.09; p > 0.5). Taken together, these findings supported our hypothesis that the increased activity in dmPFC that we isolated during the financial bubbles reflected a computation associated with the participants’ tendency to make inferences about the mental states of other players in the market. An intriguing possibility is that participants during the financial bubble, rather than mentalizing the intentions of individual players, would represent the whole market as an intentional agent in the attempt to forecast the future intentions of the market.

**Figure 5:**
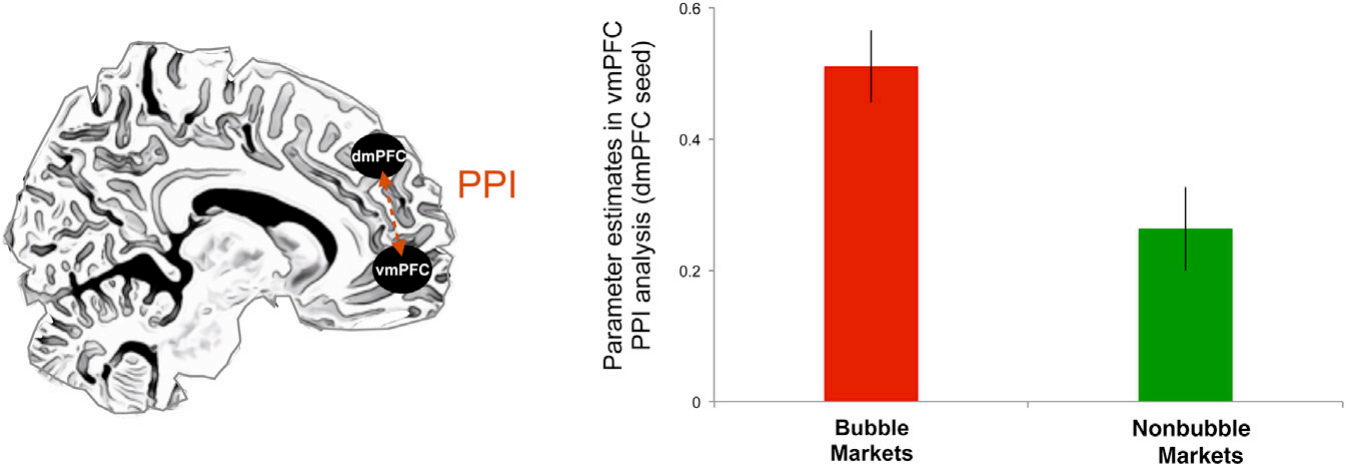
Functional Connectivity of vmPFC-dmPFC Psychophysiological interaction (PPI) analysis between dmPFC (seed) and vmPFC (target) during bubble markets. The bar plot shows how activity in vmPFC (8 mm sphere centered at −6, 50, 1) shows an increased functional coupling with dmPFC during bubble markets (p < 0.001). Error bars represent SEM.

Notably, unlike in vmPFC, activity in dmPFC isolated in this contrast did not correlate significantly (ρ = 0.009; p > 0.5) with the individual’s susceptibility to ride a financial bubble, as measured by the bubble susceptibility index. These results suggested that the neural circuit that modulated the value representation in vmPFC (associated with the behavioral susceptibility to ride a financial bubble) might be influenced by the social computations instantiated in dmPFC during the update of participants’ CPV. In order to test this hypothesis, we then conducted a psychophysiological interaction (PPI) analysis between vmPFC and dmPFC. This analysis revealed that the functional coupling between these two regions significantly increased during bubble markets (p < 0.001; Figure 5), suggesting that investors might update their portfolio profits in vmPFC by taking into account the intentions of the other players in the market. We therefore devised a model-based analysis to investigate this idea in more detail.

### Intentionality

To study how intentions modulate market traders’ computations, we studied how subjects inferred intentional agency from changes in the arrival of buy and sell orders. Recall that subjects see a fast-motion replay of all orders to buy (bids), and all orders to sell (asks), which were entered in the original behavioral experiments. Paying careful attention to this fine-grained sequence of buy and sell orders could form a basis for predicting trader intentions (a relative of sentiment in financial economics; Baker and Wurgler, 2007). To translate this idea into a precise computational variable, we use a recent precise measure from financial theory. The intuitive idea is that the presence of strategic agents in a market can be inferred by a statistical change in the order arrival process, from a homogeneous Poisson process to a mixture process (where the arrival intensity switches randomly) (Easley et al., 1997). The idea is that any increase in trader information, or even a perception of such an increase, will change order arrival. For example, orders may arrive more rapidly as traders try to trade quickly before information leaks out, or orders may thin out as traders place orders more cautiously, afraid of being on the wrong end of a trade against a better-informed partner (Easley et al., 2002).

We therefore constructed a statistic that measured the dynamic of breaks in Poisson homogeneity during trading. We called this metric Poisson inhomogeneity detector (PID). PID is a statistic that increases as the evidence against a homogenous Poisson order arrival process increases over the recent past. Specifically, it tests whether the number of arrivals in the last interval of 9 s conforms to a Poisson distribution with fixed arrival intensity. This measure, first proposed and investigated by Brown and Zhao (2002), has good statistical power (in small samples) to reject the null hypothesis of homogenous arrival in favor of the alternative that the arrival rates obtain from Poisson distributions with different arrival rates across the M intervals.

**Figure 6:**
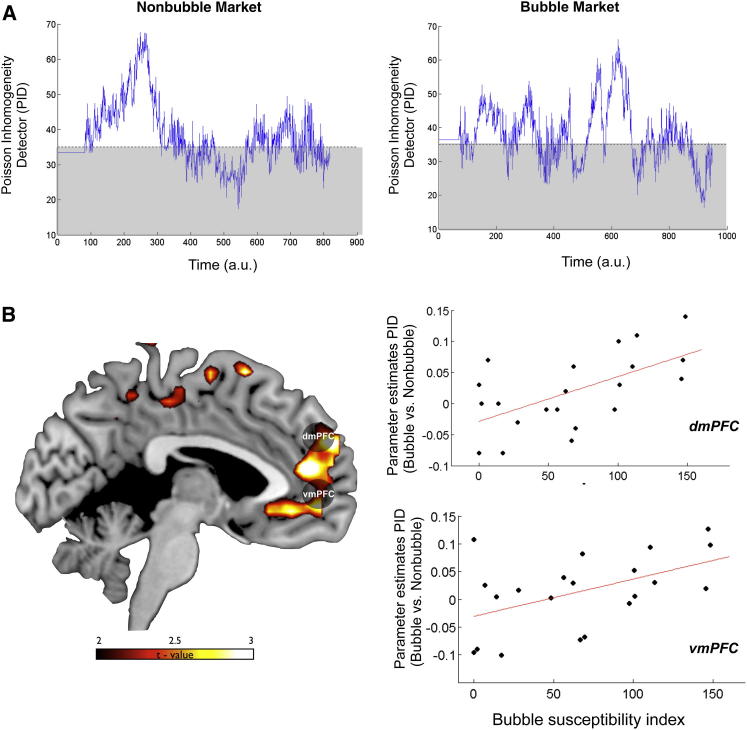
Poisson Inhomogeneity Detector Signals (A) Poisson inhomogeneity detector (PID) evolving over time for the two types of markets (bubble and nonbubble) depicted in Figures 1C and 1D. This metric captures the inferred change in evidence (at p = 0.05) for a switch from a homogeneous Poisson process in the arrival of orders (gray box) to a mixture process, in which arrival intensity changes randomly. (B) Response in medial prefrontal cortex to parametric changes in PID in bubble markets versus nonbubble markets, which is positively modulated by the individual propensity to ride a financial bubble. The scatter plot shows the parameter estimates for each participant in the dmPFC and vmPFC ROIs. The scatter plot is solely for illustrative purposes (e.g., to show the absence of outliers), and it is not used for statistical inference.

Letting $x_{i}$ denote the number of arrivals in interval i(i=1,…,M), and

(Equation 1) $$y_{i}={\left(\frac{x_{i}+3}{8}\right)}^{1/2},$$

then the PID is defined as

(Equation 2) $$PID=4\sum_{m}^{i}{\left(y\left(i\right)-Y\right)}^{2},$$

where Y equals the average (across M intervals) of the values of $y_{i}$. Under the null hypothesis, PID approximately follows a χ^2^ distribution with M − 1 degrees of freedom. Taking M = 24, this means that the critical value corresponding to p = 0.05 is PID = 36. As PID grows, the evidence against the null hypothesis of no change in arrival rate increases (Figure 6A; Figure S4).

Using this model, we were then able to construct a parametric regressor for each subject, measuring inferred intention over time. The regressor averaged the value of PID over the period in which the subject observed the arrival of asks and bids in the market (see Experimental Procedures).

Critically, this parametric regressor was uncorrelated with either CPV (r = 0.06 ± 0.02) or the deviation in prices from the fundamental values (r = 0.001 ± 0.09). Changes in PID were then input as a parametric regressor in a general linear model to test whether activity in vmPFC and dmPFC showed a greater modulation to this metric during a contrast between bubble markets versus nonbubble markets (analogously to the contrast using CPV as modulator). We then extracted the signal in both regions of interest (using an 8 mm sphere centered at [3, 53, −2] for vmPFC and [9, 50, 28] for dmPFC). This analysis yielded a significant result in both regions in medial prefrontal cortex (vmPFC: t = 1.83, p < 0.05 and dmPFC: t = 1.77, p < 0.05). We then tested how this activity in medial prefrontal cortex covaried with the susceptibility to ride the bubble (i.e., correlation with bubble susceptibility index). A significant correlation in most of the medial prefrontal cortex (Figure 6B), including the two regions of interest, vmPFC (r = 0.46; p < 0.001) and dmPFC (r = 0.68; p < 0.001), was isolated as a result of this analysis (Figure 6C; for a complete list of activations, see also Table S1).

## Discussion

Understanding why financial bubbles occur is a challenging problem that has been intensively investigated, with no clear results. Several scholars have recently started to explore the neural mechanisms underpinning human behavior during financial interactions (Knutson and Bossaerts, 2007; Kuhnen and Knutson, 2005; 2011; Lohrenz et al., 2007), along with psychophysiological (Lo and Repin, 2002) and hormonal measures (Coates and Herbert, 2008). However, nothing is known about the neural computation underpinning traders’ behavior during financial bubbles. Here, we show that neuroscientific data can help make sense of market behavior that is anomalous for standard financial theory (Yu and Xiong, 2011) by emphasizing the role played by traders’ theory of mind in artificially inflating the value of portfolio profits.

Standard asset pricing theory assumes that competitive markets are nonstrategic and nonintentional (i.e., payoffs depend only on the price, which one cannot influence). On the contrary, our behavioral results show that the explicit information carried by prices and fundamental values accounts for significantly less variance in choice behavior when subjects are trading in bubble markets. When we tested how trading in bubble markets modulated the representation of trading values in vmPFC, we showed that these values are differentially represented in vmPFC. More specifically, trading in the context of a financial bubble is associated with inflated value representations in vmPFC. Many studies show that vmPFC plays a key role in valuation and goal-directed choices (Rangel et al., 2008; Boorman et al., 2009; Chib et al., 2009; FitzGerald et al., 2009; Hare et al., 2009; Levy and Glimcher, 2012). Contextual factors have a powerful effect in modulating the neural representation of goal values in vmPFC and therefore affect choice (Plassmann et al., 2008; De Martino et al., 2009). For example, inflated value representation in vmPFC has been previously shown to affect prices, causing a behavior known as money illusion (Weber et al., 2009). This behavior is associated with vmPFC tracking the inflated nominal value even when the actual purchasing value remains unchanged.

Investigating changes in value representation in vmPFC, we were able to show a correlation between the propensity to ride a bubble (measured with the bubble susceptibility index) and activity in this region. Note that in our experiment, participants could ride the bubble, but not directly influence its formation, due to the nature of the experimental design. However, this situation is analogous to real financial markets in which the action of a single trader very rarely has a detectable impact on the whole market. We then sought to clarify the role played in this process by participants’ attempts to forecast the intentions of other players or of the market as an intentional agent.

In fact, while standard financial theory assumes that competitive markets are nonstrategic, it is not uncommon for people to assign intentionality to markets. Financial commentators often say, anthropomorphically, that “markets are panicking” or “markets are losing confidence.” Assigning intention or agency is a natural way for humans to model and interpret complex behavior (as in the case of simple societies in which human-like gods are thought to control natural processes such as the weather). Humans live in social environments and therefore usually benefit from ToM abilities that allow them to forecast the intentions of others and take preventive actions (Fehr and Camerer, 2007; Frith and Frith, 1999; Gallagher and Frith, 2003; Sanfey, 2007), an ability instantiated in medial prefrontal cortex (dmPFC) (Amodio and Frith, 2006; Frith and Frith, 2006).

Using an independent ToM task (Baron Cohen et al., 2001), we showed that the increase of activity isolated during the bubble markets correlates with the individual ability in ToM. Furthermore, we showed that the functional coupling between dmPFC and vmPFC was increased during bubble markets. We interpreted these results by proposing a putative mechanism that produces the increase in value sensitivity that we observed in vmPFC while participants traded in the context of bubble markets. These data suggest that during financial bubbles, participants are taking into account the intention of other players in the market (or of the market as whole) while updating their value estimates, and that this effect is mediated by the interaction between dmPFC and vmPFC. This interpretation fits with previous studies that have highlighted the role of dmPFC in shaping value computation by showing that social signals change the way in which values are updated through reinforcement learning (Behrens et al., 2008; Hampton et al., 2008; Behrens et al., 2009; Suzuki et al., 2012). For example, activity in dmPFC correlates with the likelihood that participants playing a “work-or-shirk” strategic game learn the value of an action using a model that takes into consideration the intentions of the other players in the game (Hampton et al., 2008). A recent study by Nicolle and colleagues (Nicolle et al., 2012) has proposed that dmPFC is not specifically involved in mentalizing but has a more general role in representing the values of actions that are modeled but not executed while vmPFC is involved in representing only those values that are relevant for the decision maker’s executed choice. According to this framework, a complementary interpretation of our results is that the activity in dmPFC reflects a computation of value associated with modeled alternative choices (e.g., buying at different prices from the fundamental value) that are especially relevant for traders during bubble markets, when the price path is highly variable.

To provide further support to the hypothesis that the attempt to forecast the intentions of other players or of the market plays a key role in modulating the susceptibility to financial bubbles, we devised a new statistic, the PID, to interrogate our neural data using a model-based approach. The rationale behind this analysis was suggested by recent financial models that have proposed that the presence of intentionality in the market (i.e., strategic agents in financial terms) can be inferred by changes in the order arrival process from a homogeneous Poisson process to a mixture process whereby orders arrive in clusters, followed by periods of unusually low activity (as if traders were holding their breath). Finance theory (Easley et al., 1997) and some experimental evidence (Camerer and Weigelt, 1991) suggest that a change in order arrival indicates the presence of traders who are better informed or who are perceived to be better informed. Therefore, the PID statistic can be considered a measure of the intensity of the perceived winner’s curse and hence of inferred intention in the marketplace. Note that even in the absence of strategic players in the market, it is sufficient that participants perceive (and believe) that there are agents with an information advantage, i.e., that there are agents who make better guesses about when a bubble may crash (Abreu and Brunnermeier, 2003). This metric allowed us to measure if activity in vmPFC and dmPFC was positively modulated during bubble markets in response to change in the level of perceived intentionality in these markets.

It is important to highlight that while the PID statistic shows fluctuations in the nonbubble markets too (primarily in the initial periods in which bids are below the fundamental value, a standard feature of all types of experimental markets), activity in these prefrontal regions specifically responds to change in intentionality (perceived or real) during the bubble markets, a type of market in which the fundamental values are not sufficient to predict the future evolution of prices.

Our analyses showed that both regions were positively modulated by the PID parameter during bubble markets and that activity in the dorsal and ventral regions of the medial prefrontal cortex showed a positive modulation with the susceptibility to ride financial bubbles. It is worth noting that the PID parameter is orthogonal to the CPV parameter used in the first analysis, so the PID analysis is likely to pick up different computational processes carried out by the same regions. Taken together, these data provide further support that forecasting intention plays a key role in modulating the regions in medial prefrontal cortex that we have identified to be involved in ToM and value computation during the representation of trading values in financial bubbles. However, the exact way in which these different computations interact to shape behavior needs to be investigated in further detail using tailored experimental paradigms. We also want to emphasize that our study does not exclude the possibility that other mechanisms (such as anticipatory affective response), which have been demonstrated to lead to financial mistakes (Wu et al., 2012, Kuhnen and Knutson, 2005), might also play a pivotal role in the formation of bubbles. Financial bubbles are complex and multidimensional phenomena, and the identification of the neural mechanisms underpinning their formation requires the combination of a number of different approaches.

In conclusion, in this study we showed how the same computational mechanisms that have been extremely advantageous in our evolutionary history (such as the one that allows people to take into account the intentions of other agents when computing values) could result in maladaptive behaviors when interacting with complex modern institutions like financial markets. However, it must be noted that these abilities are not always maladaptive in a financial milieu. For example, traders can successfully use their ToM abilities to detect the presence of insiders in the market (Bruguier et al., 2010), inducing traders to become more cautious in order to avoid being taken advantage of by a better-informed trading partner and improving the estimation of prices. Overall, our work suggests that a neurobiological account of trading behavior (Bossaerts, 2009) that takes into account theory of mind can provide a mechanistic explanation of financial concepts such as limited-rationality investing (Fehr and Camerer, 2007). The insights that this study gives into the underlying computational mechanisms that lead to bubble formation can also potentially benefit policymakers in designing more efficient social and financial institutions.

## Experimental Procedures

### Participants

Twenty-six undergraduate and graduate Caltech students took part in the original 2-day scanning study. Because of potential gender differences in financial and social behavior (Powell and Ansic, 1997; Eckel and Grossman, 2008; Byrnes et al., 1999; Bertrand, 2011), the study included males only. Five subjects were excluded from the analysis because of technical problems at the time of the scanning or excessive head movements.

### fMRI task

Trading activity in six actual experimental markets (collected in previous behavioral studies; Porter and Smith, 2003) was replayed over a 2 day scanning schedule. Three of the markets used in the study were nonbubble markets; in these markets, the market prices closely tracked the fundamental value of the asset. The other three markets were bubble markets, in which market prices rose well above the intrinsic value (see Figure S1). On each day, the participants traded in three experimental markets selected in a pseudorandom order (to avoid three consecutive markets of the same type being presented in the same day). The duration of each market was approximately 15 min. Participants started each new session with a cash endowment of $60 and zero shares. Each market was divided into fifteen trading periods. At the beginning of each period, participants were shown a message stating the period number and the value of their portfolio (shares and cash). This was followed by a video showing an intuitive graphical replay of the order (asks and bids) and trade flow. The scanned participants observed a fast-motion visual representation of the prices of offers to sell (asks) and offers to buy (bids), which were actually inputted by the participants who had taken part in the original behavioral experiments. The orders were arranged by price level (see illustrative diagram on the right corner of Figure 1). Whenever a trade occurred, the best bid (if a sale) or best ask (if a purchase) briefly (0.5 s) changed color to green, after which the circle disappeared. The circles constantly rearranged to ensure that the best bid and ask circles were closest to the midpoint of the screen (this graphical representation of the trades was a modification of an fMRI task used by Bruguier and collegues (Bruguier et al., 2010). After a variable time interval (3–6 s), the screen was frozen for 5 s, and participants used their initial endowment of $60 to either buy or sell (1, 2, or 3 shares) or stay by pressing a keypad. The intervals in which choices were made (choice intervals) were presented 2–3 times during each of the 15 periods composing each market. After the choice was inputted (5 s choice interval), an update of the participant’s portfolio (number of the shares held and cash) was presented on the screen. At the end of each period (15 periods in total for each market), a dividend was randomly extracted from a uniform distribution of (0¢ 8¢ 28¢ 60¢), and participants were then paid for the number of shares held. Participants were also allowed to short sell shares for a total maximum of 52 shares. In cases of short selling, participants had to pay the cost of the dividend for the number of negative shares held. At the end of each period, the dividend for that period was displayed to the participants with an update of their portfolio. For full instructions given to the participants’ in advance of the experiment, please see Appendix 1 in the Supplemental Information.

### ToM Task

All participants that took part in the original experiment were contacted via e-mail and asked to complete an online modification of the eye gaze ToM task (Baron Cohen et al., 2001). Seven of the twenty-one participants that took part in the original fMRI study did not respond to our request. The remaining fourteen participants who did complete the online testing received a $10 Amazon voucher as compensation. During the test, participants were shown 36 photographs of eye gazes in a consecutive sequence, and they were asked to pick one term from four possible descriptions of the person whose eyes were portrayed in the photo (for example, anxious, thoughtful, skeptical, suspicious).

### Behavioral Analyses

Behavioral analyses were performed using Matlab statistical toolbook and SPSS. Ordered logistic regression was implemented using the PLUM (polytomous universal model) procedure in SPSS (DeCarlo, 2003). The dependent variables were the participants’ choices coded as trinary variables (i.e., buy, sell, or stay), while the two dependent measures were market prices (average of best bid and best ask available in the choice period) and fundamental asset value for the current period ($0.24 × [15 − t + 1]) (dashed line in Figures 1C and 1D). For each model, we reported the Nagelkerke pseudo R^2^ (Nagelkerke, 1991) and the BIC (Kass and Raftery, 1995).

### Scanning Acquisition

Forty-five slices were acquired on a 3T Siemens Trio at a resolution of 3 mm × 3 mm × 3 mm, providing whole-brain coverage. A single-shot echo planar imaging (EPI) pulse sequence was used (TR = 2800 ms, TE = 30 ms, FOV = 100 mm, flip angle = 80°). The images were collected at a tilted angle of 30° from the anterior commissure. For each subject, at the end of the first scanning day (day 1), the EPI functional scanning was followed by a whole-brain, high-resolution, T1-weighted anatomical structural scan and local field maps.

### fMRI-SPM Analyses

Image analysis was performed using SPM8 (http://www.fil.ion.ucl.ac.uk/spm/). The first five volumes from each session were discarded to allow for T1 equilibration. Raw functional, structural, and field map files were reconstructed using TBR. Field maps were reconstructed into a single-phase file. This field map file was then used to realign and unwarp EPI functional images. Structural images were reregistered to mean EPI images and segmented into gray and white matter. These segmentation parameters were then used to normalize and bias correct the functional images. Normalized images were smoothed using an 8 mm full-width Gaussian kernel at half-maximum (FWHM). A GLM was constructed in which onset regressors (beginning at the start of each video) for each session were assembled by convolving δ functions with a canonical hemodynamic response function (HRF). These regressors were modulated by a parametric regressor coding for the CPV, a combination of the value in cash and in shares held by a subject at each point in time (CPV = cash + [shares × fundamental value at time t]). A correction for temporal autocorrelation in the data (AR 1 + white noise) was applied. Finally, six motion parameters were included in the GLM. In order to find an interaction of the increased value representation due to the bubble manipulation, we contrasted linear increase to CPV in the bubble markets versus the nonbubble markets.

To test the role of ToM in dorsomedial prefrontal cortex, we extracted activity from an 8 mm sphere region of interest (ROI) centered in dmPFC [9, 50, 28] isolated in the whole brain SPM analysis. We then tested how activity that parametrically tracked the increase in CPV correlated with individual ToM scores during bubble markets and nonbubble markets, calculating Spearman’s rank correlation coefficient between the parameter estimates in dmPFC and ToM scores. For the analysis using the PID, we calculated this metric (as described in the Results) for each time point in the original markets used as stimuli for the fMRI study. We then averaged the PID over the period of movie observed by each participant and used this parameter in a new GLM. We then contrasted this parametric regressor in the bubble markets versus the nonbubble markets and extract activity of two ROIs of 8 mm sphere centered in dmPFC [9, 50, 28] and vmPFC [3, 53, −2].

### fMRI-PPI Analysis

To assess changes in connectivity between dmPFC and vmPFC as a function of the market type, we carried out a psychophysiological interaction (PPI) analysis. PPI is a measure of context-dependent connectivity, explaining the regional activity of other brain regions (here vmPFC) in terms of the interaction between responses in a seed region (here dmPFC) and a cognitive or sensory process. We carried out PPI analysis using the generalized PPI toolbox for SPM (gPPI; http://www.nitrc.org/projects/gppi). gPPI creates a new GLM in which the deconvolved activity of the seed region (8 mm sphere centered in dmPFC [9, 50, 28]) is assigned to the regressors modeling the effect of the task at the time of the trading periods and reconvolved with the hemodynamic response function. Average time courses were extracted from all voxels within an 8 mm sphere surrounding the vmPFC peak coordinate [3, 53, −2] that we isolated in the original SPM analysis. This was done since the aim of this analysis was to demonstrate that the activity we isolated in dmPFC and vmPFC (in the main SPM contrast) showed a functional connectivity. The main effects of the task, seed region time course, and motion parameters were included as regressors of no interest. The PPI contrast compares bubble markets (+1) with nonbubble markets (−1).

### Statistical Inference

Second-level group contrasts from our GLM were calculated as a one-sample t test against zero for each first-level linear contrast. Activations were reported as significant if they survived familywise error correction (FWE) for multiple comparisons across a volume of 8 mm (SVC) cantered on peak of activity isolated in independent studies. For vmPFC, we used the coordinates [0, 53, 4] taken from (Suzuki et al., 2012); for dmPFC, we used the coordinates [−3, 51, 24] taken from (Hampton et al., 2008).

## Figures and Tables

**Table 1:** Ordinal Logistic Regression

Market Parameter	Bubble Markets	Nonbubble Markets
Prices	−0.011 (±0.002)^∗^	−0.020 (±0.004)^∗^
Fundamental values	0.009 (±0.001)^∗∗^	0.02 (±0.004)^∗∗^

Summary Statistics	Bubble Markets	Nonbubble Markets

Pseudo R^2^	0.27	0.33
Bayesian information criterion (BIC)	2,089	1,840

The dependent variable is an ordered variable (buy, stay, sell). The SEM is reported within parentheses; bubble versus nonbubble markets: ^∗^p < 0.05; ^∗∗^p < 0.001.
