# In the Mind of the Market: Theory of Mind Biases Value Computation during Financial Bubbles

**DOI:** 10.1016/j.neuron.2013.11.002

**Published:** 2013-11-20

**Authors:** Benedetto De Martino, John P. O’Doherty, Debajyoti Ray, Peter Bossaerts, Colin Camerer

(Neuron *79*, 1222–1231; September 18, 2013)

On page 1226, Equation 1 contained an error. In the originally published version of this article, *x*_*i*_ was added to 3 and the result was divided by 8. Instead, *x*_*i*_ should be added to 3/8. The equation has been corrected online and is shown here.

(Equation 1) $$y_{i}={\left(x_{i}+\frac{3}{8}\right)}^{1/2}$$

