# Supplementary material for: In the Mind of the Market: Theory of Mind Biases Value Computation during Financial Bubbles
Source: Neuron. 2013 Sep 18;79(6):1222–31. doi: 10.1016/j.neuron.2013.07.003 (PMC3781325; doi:10.1016/j.neuron.2013.07.003)
Supplement: Document S1. Appendix 1, Figures S1–S4, and Table S1 [file mmc1.pdf]

Neuron, Volume *79*

Supplemental Information

In the Mind of the Market: Theory of Mind Biases Value Computation during Financial Bubbles

Benedetto De Martino, John P. O’Doherty, Debajyoti Ray, Peter Bossaerts, and Colin Camerer

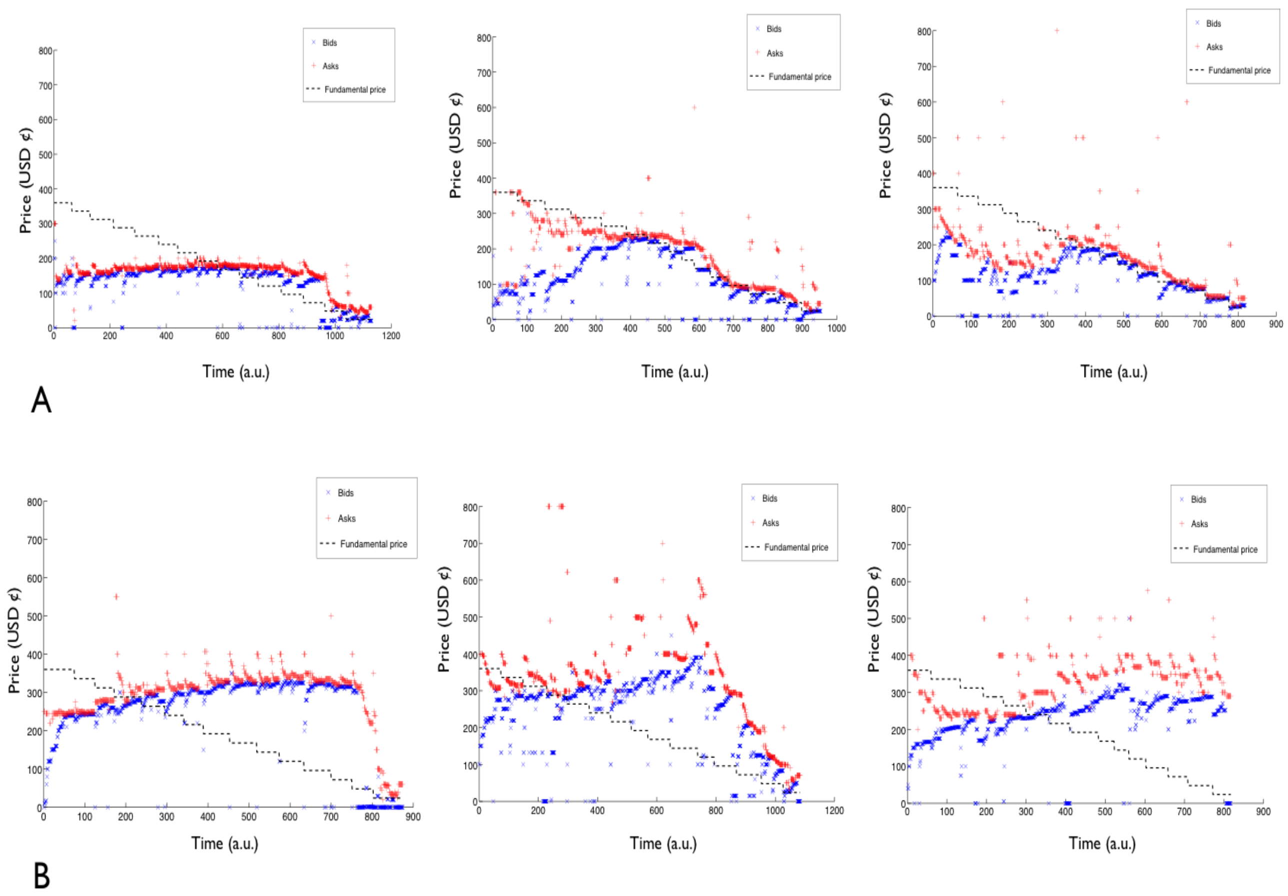

Figure S1. Related to Figure 6

Stimuli: Asks (red) and Bid (blue) plotted against the fundamental prices (dotted line) for the 3 non-bubble markets (A) and the 3 bubble markets (B) replayed during the fMRI experiment. In the non-bubble markets condition (A) asks and bids track the fundamental price over time, while in the bubble markets condition (B) asks and bids deviate from the fundamental prices.

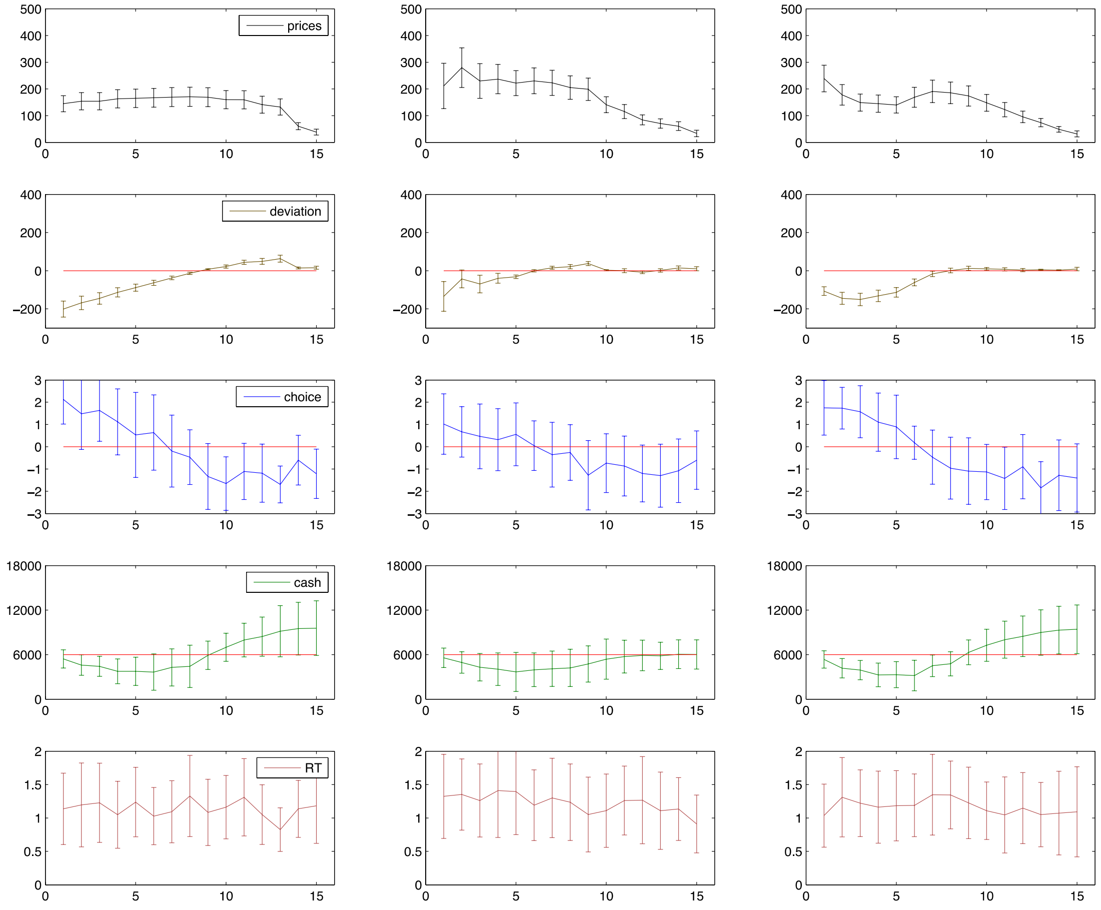

Figure S2. Behavioral data (Nonbubble Markets), Related to Results

Evolution of prices (1st row) and the deviation from the fundamental prices (2nd row – red line represent the fundamental value) affect a number of behavioral parameters: subject choice (3rd row) to buy one or more shares (positive numbers) or sell them (negative numbers); cash earned or lost (4th row – red line represent the initial endowment of $60); reaction time (5th row). The x-axis represent the 15 time periods.

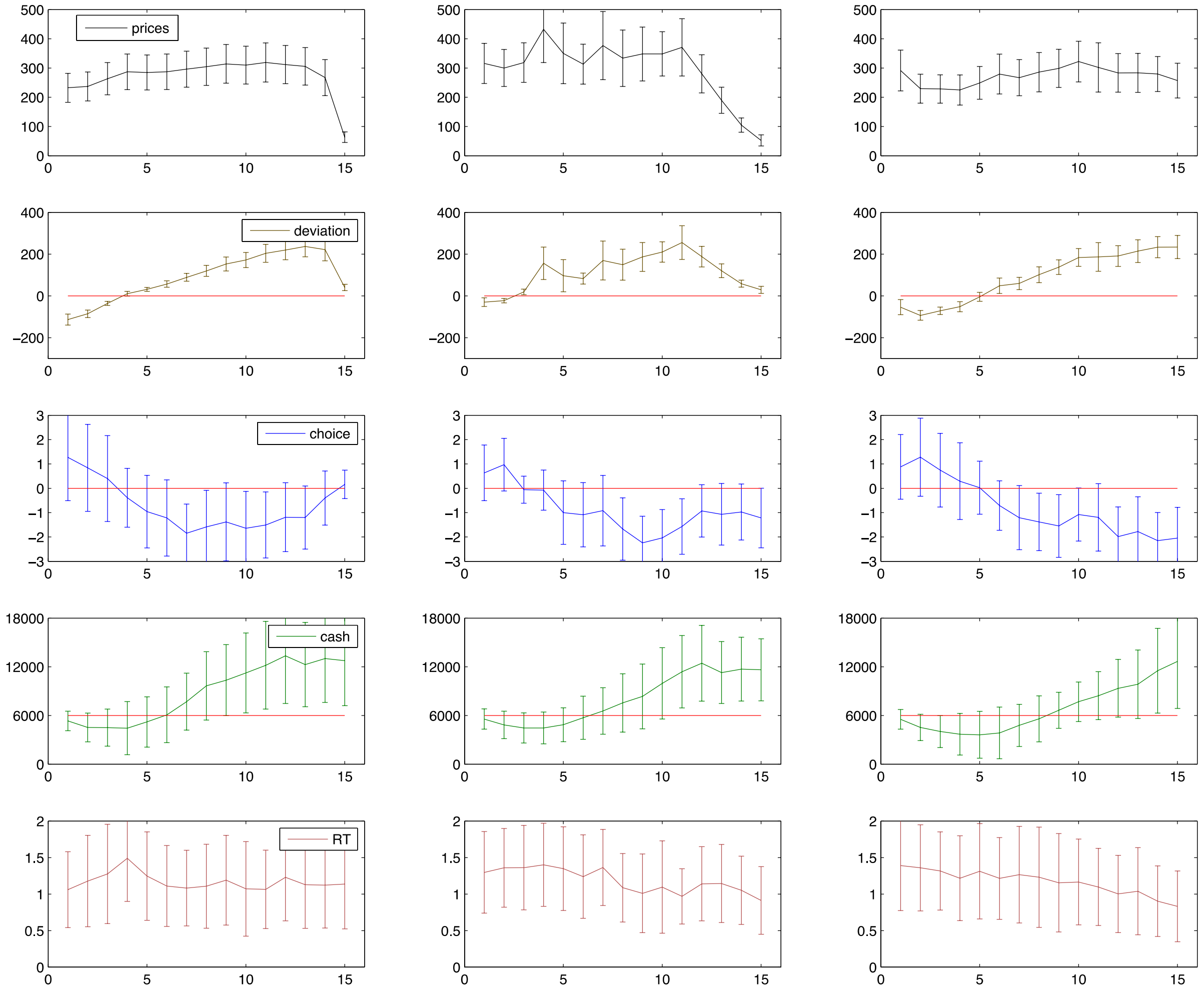

Figure S3. Behavioral Data (Bubble Markets), Related to Results

Evolution of prices (1st row) and the deviation from the fundamental prices (2nd row – red line represent the fundamental value) affect a number of behavioral parameters: subject choice (3rd row) to buy one or more shares (positive numbers) or sell them (negative numbers); cash earned or lost (4th row – red line represent the initial endowment of $60); reaction time (5th row). The x axis represent the 15 time periods.

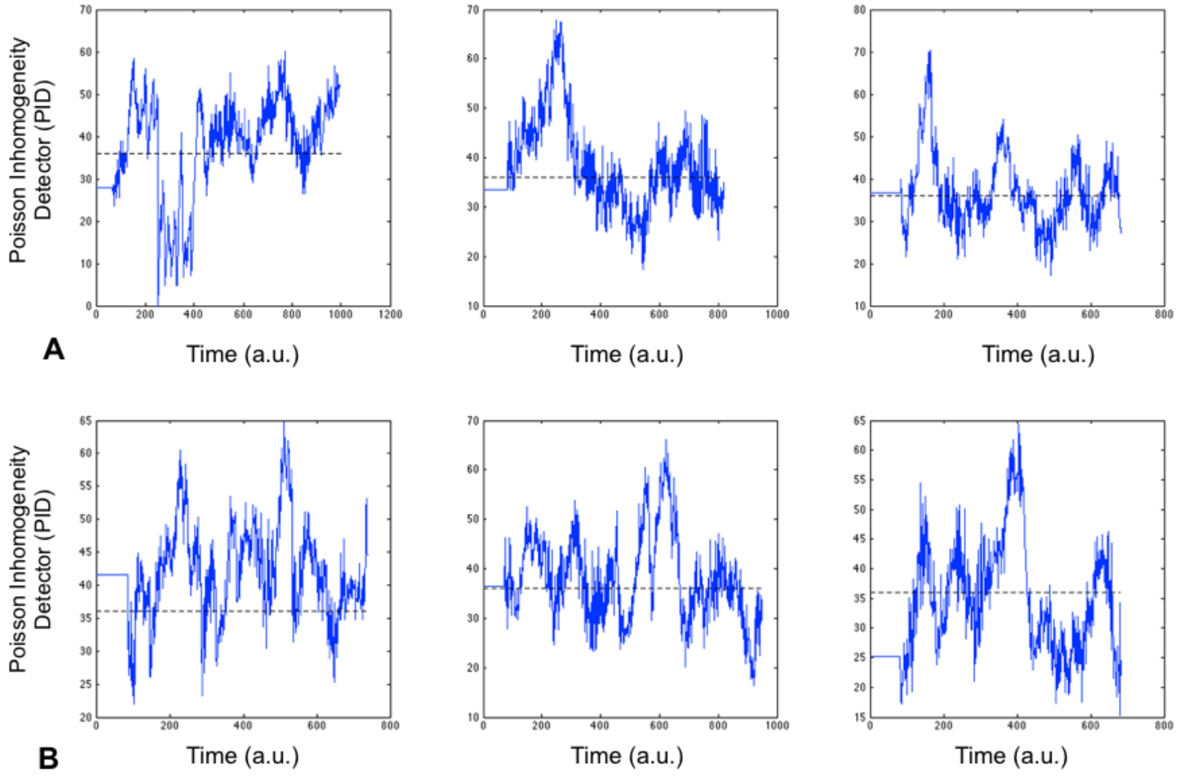

Figure S4. Related to Figure 6

The Poisson Inhomogeneity Detector (PID) evolves over time in non-bubble (A) and bubble markets (B) This metric capture the change over time the changes over time of from an homogeneous Poisson process in the arrival of orders to a mixture process (where the arrival intensity switches randomly). The dashed line represent the critical value of PID = 36 after which the evidence against the null hypothesis (homogeneous Poisson arrival) is smaller than 5% (p =0.05)

Table S1. BOLD Activations Tables, Related to Figures 2A, 3A, and 6B

CPV (Bubble Markets vs. Non-bubble Markets)

Region	MNI [x, y, z]	K_E	Z score (peak level)
Ventromedial Prefrontal cortex (vmPFC)	[3, 53, -2]	14	3.02*
	[3, 32, -14]	6	2.78
Dorsomedial Prefrontal cortex (dmPFC)	[9, 50, 28]	105	3.44*
	[15, 56, 13]	20	3.62
Pre-cuneus	[-6, 43, 49]	106	3.89
Left –Temporoparietal junction (TPG)	[-48, -52, 25]	31	3.16
Right – Superior frontal sulcus	[21, 38, 37]	105	3.60
Right – Inferior frontal sulcus	[48, 41, 4]	69	3.72

CPV (Non-bubble Markets vs. Bubble Markets)

No activations detectable at p<0.005

CPV (Bubble Markets vs. Non-bubble Market)
Covariate with Bubble Susceptibility Index – Increase

Region	MNI [x, y, z]	K_E	Z score (peak level)
Ventromedial Prefrontal cortex (vmPFC)	[-6, 50, 1]	13	3.00*

CPV (Bubble Markets vs. Non Bubble Markets)
Covariate with Bubble Susceptibility Index – Decrease

Region	MNI [x, y, z]	K_E	Z score (peak level)
Left –Superior Temporal Sulcus	[-54, -43, 16]	8	2.96

K_E = cluster size ; * = p<0.05 FWE small volume corrected

Poisson homogeneity test (Bubble Markets vs. Non-bubble Markets)
Bubble Susceptibility Index – Increase

Region	MNI [x, y, z]	K_E	Z score (peak level)
Ventromedial Prefrontal cortex (vmPFC)	[6, 41, -8]	34	3.27*
Dorsomedial Prefrontal cortex (dmPFC)	[0, 56, 28]	66	2.88*
Pre-cuneus	[-6, 43, 49]	106	3.89
Left –Central sulcus	[-27, -31, 52]	98	4.28
Right – Central sulcus	[21, -37, 52]	6	2.82

Poisson homogeneity test (Bubble Markets vs. Non Bubble Markets)
Bubble Susceptibility Index – Decrease

Region	MNI [x, y, z]	K_E	Z score (peak level)
Left– Inferior frontal sulcus	[36, 29, 28]	6	2.96

K_E = cluster size ; * = p<0.05 FWE small volume corrected

Appendix 1. Participants' Instructions, Related to Experimental Procedures

Setup

In this experiment we will replay several real securities markets that have been executed by real subjects in past experiments. Full details of these previous experiments will be given to you, upon request, at the completion of the study. You will see the history of the order flow (i.e., buy and sell offers) and the trades as they happened. We are replaying each market 10 times faster than the original experiments. Your actions will not have any effect on the history. However, you incur the same risk as some players did in the market – at given moments you will be asked to take action upon the current state of the market. The types of securities in the market are stocks that can be traded and that pay random dividends at the end of each period. (more details below)

The whole experiment will last 3 days. The first 2 days you will play 3-4 sessions (i.e. markets) while you are scanned for an average estimated time of 1 hour and a half. Every session will last about 14-18 minutes. The 3rd day you will be asked to come back to fill-in questionnaires and receive your payment. For the entire study you will receive a show-up fee of $60. However, depending upon your performance in playing the task you can make additional earnings of up to about $200.

What is the structure of a session?

Each session starts with an initial message in which we show you the period number that is about to start, the amount of money you have, and the number stocks you own. There are 15 periods for each session; you will be given $60 at the beginning of a session; and you have 0 shares to start. After this initial screen you will be shown a video of the market for a variable time (3 to 6 seconds). The video will freeze for 5 seconds and you will be asked to input your choice by key-press. You will find more details about the task below. Once you have made your choice a screen will show the update of your earnings in dollars and the number of shares you hold. We will then continue to replay the video (i.e. actual market) from the point at which it was stopped. The procedure will be repeated accordingly.

What do the videos represent?

We replay the history of the offers and the trades played by real subjects in previous experiments. A typical screen is reproduced below. Each bubble represents a current standing order. Blue bubbles are buy orders and red bubbles are sell orders. The number in the bubble represents the price of 1 share in cents. For example, the blue bubble with the number "200" stands for an order to buy at $2, while if the number was 10 the share would cost $0.10. Only offers for the best price levels are shown. There could be more offers at inferior prices. Orders with higher price levels are always above those with lower price levels. Note that a trade happens every time an order is fulfilled (i.e., a subject buys a stock from another subject at the best available price). You will realize that a trade has occurred when the bubble with that order turns green before disappearing from the screen. Note that bubbles may also disappear when market players cancel orders. On the left side of the screen is shown how much time is left until the end of the session. Note that this is the original time and the experiment is replayed 10 times faster. For example, 48:12 means that the market will finish in 4 minutes and 8 seconds. We also show you which period you are playing and the dividend of the previous period (it will be more clear what dividends are soon).

How do I play the task?

At the beginning of each session you receive $60 cash to play the market. Every time the video stops and the message "CHOOSE" appears on the screen, you are asked to take an action on the current state of the market. More specifically you can buy or sell shares at the best prices standing at that moment. These are the two prices closer to the cross in the middle of the screen. For example, in the figure above the best selling offer is $4/share (400 inside the red bubble) while the best buying offer is $2/share (200 inside the blue bubble). You can buy or sell up to 3 shares every time, or you can decide to not take any action (i.e. neither sell nor buy) by pressing a specific button. You will be instructed about the configurations of the buttons at the time of the experiment. So, in the previous example, if you decide to buy 2 shares you will pay $8. Once you make your choice the screen will show the update of your current state. In the previous example your new cash balance will be updated to $52 (i.e., $60 - $8) and your share balance to +2. Alternatively, you could have decided to sell a share for the best buying offer ($2 in the previous example) in this case your balance would have changed as follow: cash $62 (i.e., $60+2) and your share balance to -1. As you see from this example you can sell a share

even if you don't own it (for example at the beginning of the session), and your share balance will become negative. Note you have a lower limit of -50 shares that you can do down to.

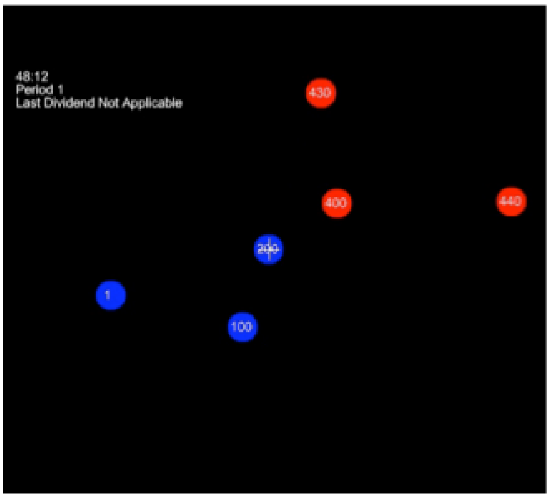

How do I make money?

You make or lose money by trading shares and by the dividend of the share you hold. Imagine you are you are a trader in Wall-street who buys or sells the shares of Apple. You may buy shares at a low price at the beginning of the year, sell them at a higher price later on in the year. Another way to make money is when Apple pays out dividends to the shareholders at the end of the year. So, depending on how well Apple did that year (for example by introducing a successful iPhone) the company will pay out a good dividend for each share.

In our experiment you will play a number of sessions or markets. Every market is divided into 15 periods at the end of which the dividend will be extracted randomly from 4 possible dividends: 0, 8, 28, or 60 cents with equal probability. Going back to the previous example, let's say that the period ends and the dividend extracted for that period is 60 cents. In the first example you bought 2 shares, so you make $1.2 (60 cents x 2). In the next period let's suppose you accumulate 12 shares. The dividend extracted at the end of this period is 28 cents. You will make $3.36 at the end of period 2. You can also sell some of the shares you hold at the best available offer price and make money by doing so.

Previously, we mentioned that you are also allowed to sell shares you don't own for a lower limit of -50 shares. If your share balance is negative e.g. -1, and the dividend extracted at the end of the period is 60, we would also multiply the dividend with the number of shares. Since this is a negative number you will need to pay $0.6 (60 cents X -1), so $0.6 will be subtracted from your cash balance. If you hold a negative numbers of shares it is like paying the other shareholder back.

What happens at the end of the session?

You will play every session (market) for 15 periods and at the end of every period dividends are paid. Remember that dividends will be extracted randomly from 4 possible dividends of 0, 8, 28, 60 cents with equal probability. So expected dividend payoff per share per period is $0.24 [i.e. (0 + 8 + 28 + 60)/4]. However, at the end of the experiment the market wills close and the shares you hold at the end are worth nothing, the only thing that counts is how much money you made by that time. This is true for both positive and negative numbers of shares. So if you have 10 shares left, they won't pay you anything back and you won't be able to sell them anymore. Similarly if you are left with a negative number of shares you won't need to pay anything back. It is as if Apple went bankrupt and is not present anymore in the stock market, so you won't get any money from the positive shares you hold, but you will not need to pay the other shareholders for the negative share you hold. Given this design, at every period, the expected future payoff for the shares you hold decreases until the end of the session, at which point the shares are not worth anything. For example a share that you buy at the beginning of the experiment (i.e. period 1) as a theoretical expected payoff of $3.6 since it is going to pay dividend for the remain 15 periods (i.e. $0.24 X 15). On the contrary a share that you buy at period 12 will value $0.96 since it is going to pay dividends only for the left 4 periods (i.e. $0.24 X 4) until the end of the session when the market close.

After you finish a whole session (about 16 minutes) you will rest for few minutes in the scanner and we will start the next session. Now you start for scratch again: you will get other $60 and 0 share. Note that you won't carry

over stocks from the previous session. This new session is in fact a different market played by different subjects in a different past experiment that we will replay for you in the scanner. Following our previous analogy it is a bit like you now trade stock of a different company, let's say Pepsi, that has nothing to do with Apple Computers you where trading before. In the two day that the experiment last you will play a total of 6 different sessions and your final earning will be the average of the earning you made in each session you played.

A final note: don't worry if you feel a bit confused right now with all this information, you will play an entire session for practice purpose before we start scanning to make sure it is all clear. Also, please don't hesitate to ask us questions if there are aspects of the task which are not clear to you.
